# The anatomical problem posed by brain complexity and size: a potential solution

**DOI:** 10.3389/fnana.2015.00104

**Published:** 2015-08-20

**Authors:** Javier DeFelipe

**Affiliations:** Laboratorio Cajal de Circuitos Corticales (Centro de Tecnología Biomédica: UPM), Instituto Cajal (CSIC) and CIBERNEDMadrid, Spain

**Keywords:** neuron doctrine, electron microscopy, connectome, synaptome, choice of species for studying the brain, interdisciplinary approaches

## Abstract

Over the years the field of neuroanatomy has evolved considerably but unraveling the extraordinary structural and functional complexity of the brain seems to be an unattainable goal, partly due to the fact that it is only possible to obtain an imprecise connection matrix of the brain. The reasons why reaching such a goal appears almost impossible to date is discussed here, together with suggestions of how we could overcome this anatomical problem by establishing new methodologies to study the brain and by promoting interdisciplinary collaboration. Generating a realistic computational model seems to be the solution rather than attempting to fully reconstruct the whole brain or a particular brain region.

## The Magnitude of the Problem

“Gentlemen, instead of promising to satisfy your curiosity about the anatomy of the brain, I intend here to make the sincere,  public confession that this is a subject on which I know nothing at all.”

—Opening words of the “Discours sur l’anatomie du cerveau” delivered by Nicolaus Steno (1638–1686) in 1669 (Steno, 1669).

**Figure 1:**
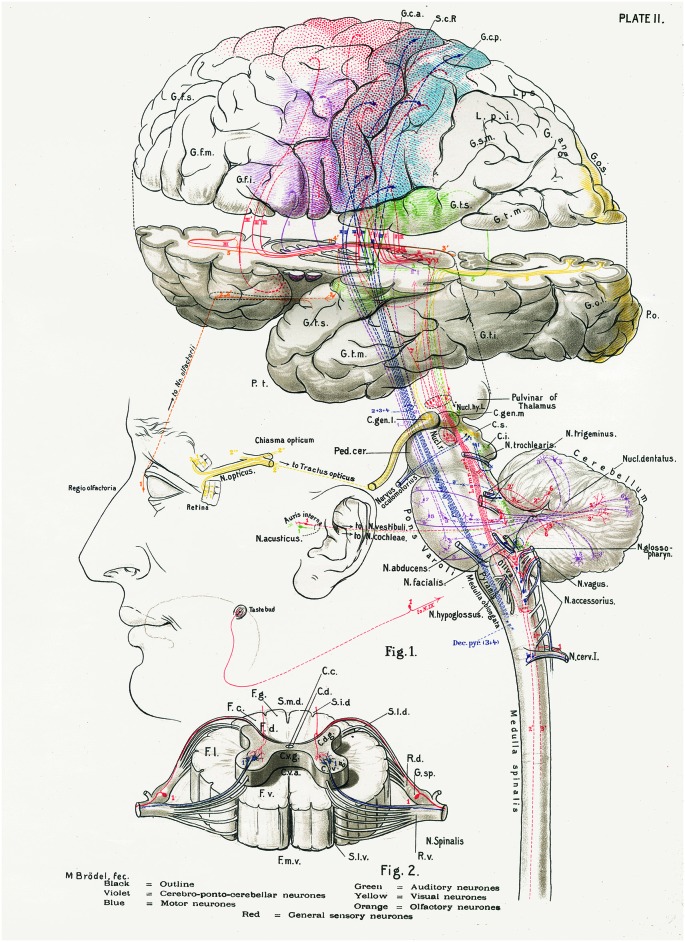
**The central nervous system works as a whole**. Schematic drawing by Barker (1899) to illustrate some of the multiple relationships between different parts of the central nervous system. Taken from DeFelipe (2014).

The words used by Nicolaus Steno to make his point could be taken as an introductory sentence to describe the magnitude of the problem in dealing with the anatomy of the brain, not only due to the complexity of its organization, but also because our knowledge of the brain is far from complete. The central nervous system works as a whole (Figure 1), and it is well established that the principles of structural design (spatial distribution, number and types of neurons, and synapses per volume, etc.) differ considerably in the different parts of the nervous system, as well as between species and strains. There is also considerable variation associated with age. Indeed, the early postnatal brain is structurally and functionally different from adolescent, young adult and older brain (Jacobs and Scheibel, 1993; Kolb et al., 1998; Marner et al., 2003; Stark et al., 2007; Feldmeyer and Radnikow, 2009; Workman et al., 2013; Luebke et al., 2015). Moreover, there is great interindividual variability in brain size, cortical thickness, number of cells, differences in dendritic trees, etc. (Jacobs et al., 1993; Uylings et al., 2005; Caspers et al., 2006; DeFelipe, 2011), as well as gender differences in multiple regions of the brain (Jacobs et al., 1993; Cahill, 2006; Alonso-Nanclares et al., 2008; Jazin and Cahill, 2010; Luders and Toga, 2010; Semaan and Kauffman, 2010). Therefore, the data obtained in one structure will not necessarily be applicable to another and thus, molecular, genetic and anatomical patterns must be examined separately in particular regions, species and strains, and for different ages and genders. When considering the magnitude of the problem further, solely from the neuroanatomical point of view, we must bear in mind the following considerations. Bota et al. (2003) suggests that in the mammalian central nervous system there are around 500–1000 different gray matter regions (e.g., the retina, dorsal lateral geniculate nucleus, and primary visual cortex); 2500–5000 neuron classes (e.g., retinal photoreceptors, bipolar cells, and ganglion cells); and 25,000–100,000 macroconnections between neuron classes (e.g., from retinal ganglion cells to dorsal lateral geniculate). The neuroanatomical information currently available in the literature provides data about 10% of all the possible long-range projections between the roughly 500 brain regions identified in the rat (Bota and Swanson, 2007). In addition, the vast majority of these studies only provide a qualitative vision of the projections. Thus, we are very far from obtaining a quantitative connectome map. In fact, we do not yet even have a *complete* map, let alone a quantitative one. For example, since the seminal study of Felleman and Van Essen (1991) of the cortical projections to areas V1, V2 and V4 in the primate cerebral cortex, there has been a major increase in the number of areas reported to project to these areas (see e.g., Markov et al., 2011). As we will see below, this problem is several orders of magnitude higher when we consider the information available using electron microscopy and indeed there is virtually no quantitative electron microscopy data.

## Choice of Species for Studying the Brain

**Figure 2:**
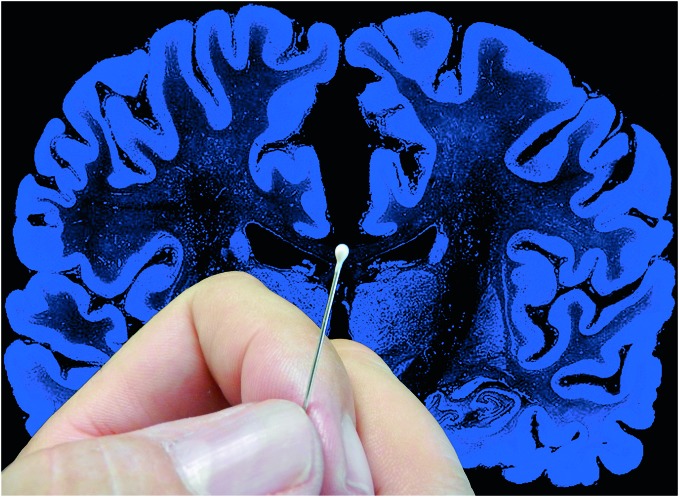
**The complexity of the brain**. Artistic composition showing a coronal histological section of the human brain and a hand holding a pin with a pinhead (approximately 1 mm^3^) to graphically illustrate the complexity of the brain. In a volume of human cerebral cortex similar to the pinhead in this figure, there are about 27,000 neurons and 1000 million synapses (Alonso-Nanclares et al., 2008). The diameter of the pin (0.5 mm) is equivalent to the thickness of a cortical column. Since a human pyramidal neuron typically has a dendritic tree with a minimum total length of several mm, in this volume there would be several thousand mm of dendrites. Taking a medium-sized pyramidal neuron with a dendritic length of 10 mm as an example, and considering that pyramidal cells represent approximately 80% of the total population (see text for further details) there would be approximately 216 m of pyramidal cell dendrites in this 1 mm^3^. Furthermore, the brain is one of the organs of the body with the highest metabolic demands and thus, there is a very dense network of blood vessels in association with the neurons and glia (see e.g., Blinder et al., 2013; Magistretti and Allaman, 2015; Yuan et al., 2015). Taken from DeFelipe (2014).

Understanding the human brain is the ultimate goal but this is extremely challenging—not only because of its complexity (Figure 2) and the technical difficulties involved, but also because ethical limitations do not allow all of the necessary datasets to be acquired directly from human brains. Consequently, most of our present knowledge of brain structure and behavior has been obtained from experimental animals. The problem is that data from nonhuman brains cannot fully substitute information on humans since there are fundamental structural and behavioral aspects that are unique to humans as well as to any other species (see e.g., Oberheim et al., 2009; DeFelipe, 2011; Sherwood et al., 2012; Geschwind and Rakic, 2013; Kaas, 2013; Hofman, 2014; Rilling, 2014). Accordingly, the question remains as to how much of this nonhuman brain information can be reliably extrapolated to humans, and indeed it is important to establish what the best strategy currently is for obtaining the missing data.

It is a common belief that the use of large mammals, like monkeys or cats, as experimental animals gives more information about the human brain than would be obtained by using small mammals, like mice or rats. Some scientists propose that research using nonhuman primates is essential for bridging a hypothetical gap between the mouse and the human brain (see e.g., Geschwind and Rakic, 2013; Homman-Ludiye and Bourne, 2014). However, there is no bridge between brains; all species have different brains and we will never fully understand how the human brain is functioning by understanding, for example, how the macaque brains do. Certainly, the study of the brain of nonhuman primates has provided us with some very valuable insights about the functional organization of brain regions where generalizations can be made (see e.g., Passingham, 2009). Indeed, comparisons are useful if we want to study certain attributes that humans alone share with certain species—like the ocular dominance columns or orientation columns of the visual cortex, which are present, for instance, in monkeys and cats but not mice and rats (Horton and Adams, 2005). It is also interesting to observe that there are clear differences in the cyto- and myeloarchitecture and chemical neuroanatomy of the thalamus between different mammalian species, indicating differences in their synaptic organization (Jones, 2007b). For example, GABAergic neurons are very numerous in the ventrobasal nucleus of the thalamus of cats, monkeys and humans, but are absent in mice and rats (Arcelli et al., 1997). Thus, macaques, for example, may be considered “more similar” to humans than rodents in this respect. However, from the microanatomical and neurochemical points of view, their brains show many important differences compared to humans, which probably reflects the obvious cognitive differences between macaques and humans (Elston, 2003; Raghanti et al., 2010b; DeFelipe, 2011). For instance, there are remarkable differences between humans and other species in various aspects of the patterns of cholinergic, serotonergic and dopaminergic innervation of the cerebral cortex, like significant differences in the density and laminar distribution of fibers expressing these neurotransmitters (for a review, see Raghanti et al., 2010a). A good example is that, in humans and other primates, all cortical areas receive dopaminergic inputs, whereas in rodents there is little or no dopaminergic innervation in many cortical areas (e.g., the motor, premotor, and supplementary motor areas, and the parietal, temporal, and posterior cingulate cortex; see Berger et al., 1991). This is important to keep in mind since these neurotransmitters exert modulatory effects on a variety of cognitive functions and are involved in many neuropathologies, such as Parkinson’s disease, Alzheimer’s disease, depression, schizophrenia and drug addiction. In other words, these differences probably indicate evolutionary adaptations to particular functions. Thus, the functional significance of the human-specific structure should be dealt with by employing a range of specific strategies.

One such strategy is the creation of a “complete” Brain Atlas, which will be particularly useful for better understanding brain structure and function as we will be able to integrate key information about the human brain, including multi-scale anatomy [macroscale (mm), microscale (μm) and nanoscale (nm) data collection] and data from fMRI, MRI, DTI and MEG. This integrative atlas will also be an excellent tool to study brain diseases by integrating clinical data (Toga et al., 2006). Nevertheless, it should be noted that most studies on human brain structure have been carried out at the light microscopic level and for good reason; studying the human brain via electron microscopy techniques presents certain problems. The scarcity of human brain tissue that is suitable for the study of synaptic circuitry is one of the most important issues to be overcome. Autopsy may be the sole source of control tissue (i.e., from individuals without known psychiatric illnesses or brain pathologies). Since there can be a long delay between death and tissue collection (often over 5 h), the ultrastructure of the post-mortem brain tissue is generally not well preserved, which makes the tissue unsuitable for detailed quantitative analysis. This is one of the principal reasons why synaptic circuitry data for the normal human brain is so lacking. Thus, a major goal would be to use human brain tissue with post-mortem times of less than 2 h and improve the current technologies for the microanatomical analysis of the human brain by adapting methodologies that are normally used to examine the brain of experimental animals. One example of this would be improving the ultrastructural preservation of autopsy human tissue using microwave oven fixation and recently developed automated electron microscopy techniques (see e.g., Blazquez-Llorca et al., 2013; Kuwajima et al., 2013).

By contrast, the human brain shares many common features with other nonhuman mammals that might be considered as basic bricks of brain organization which by definition are common to all mammalian species. Therefore, choosing appropriate experiments to obtain strategic data that could be extrapolated to the human brain will be another major goal. For this general purpose, many neuroscientists suggest that the ideal experimental animals at present are rodents because they can be manipulated to study many aspects from genes to behavior. Furthermore, we can use relatively large numbers of animals at a relatively low cost. What follows is a discussion of the further problems that we have to face from the anatomical point of view, based on a previous publication of the author (see DeFelipe, 2010). An attempt will also be made to clarify frequent misunderstandings and wrong assumptions about certain aspects of the brain organization.

## Starting Point: The Neuron Doctrine

*The great enigma in the organization of the brain revolves around our need to ascertain how the nervous ramifications end and how neurons are mutually connected. Referring to a simile already mentioned, the idea was to inquire how the roots and branches of the trees in the gray matter terminate, so that in such a dense jungle, in which there are no gaps thanks to its refined complexity, the trunks, branches and leaves touch everywhere*.

—Cajal (1917, p. 100; Recuerdos de mi vida)

Our schemes of how neurons function and interchange information with other neural elements is based on the central principle of neuroscience established by the Neuron Doctrine, that is, that the neuron is an independent cellular unit with an overall polarization that mediates its input-output functions (Shepherd, 1991; Jones, 1994). In general, neurons can be divided into distinct morphological and functional regions: a receptor apparatus (formed by the dendrites and cell body or soma), the emission apparatus (the axon), and the distribution apparatus (terminal axonal arborization).

Nevertheless, there are many exceptions that challenge the neuron doctrine. Concerning synaptic relationships, axons have been found to form synapses with other axons and presynaptic elements can be dendrites or somata. Thus a variety of synapses exist in addition to the “classical” axo-dendritic and axo-somatic synapses: axo-axonic, dendro-dendritic, somato-somatic, somato-dendritic, dendro-somatic, dendro-axonic and somato-axonic synapses (Peters et al., 1991). Furthermore, neurons are not only connected by point-to-point chemical synapses, but may also be coupled electrically, and the direction of transmission may be bidirectional through small channels known as gap junctions. A gap of approximately 2 nm separates the plasma membranes of neighboring neurons, and it is the presence of these gap junctions that allows the diffusion of small molecules as well as the flow of electric current (Bennett, 2000; Bennett and Zukin, 2004). Electrical interaction also occurs between neurons that are in very close proximity, even in the absence of specialized membrane structures (Anastassiou and Koch, 2015). Moreover, the transmitter released at synaptic or non-synaptic sites may diffuse and act on other synaptic contacts, or on extrasynaptic receptors (Fuxe et al., 2007). It is also known that neuromodulators (e.g., serotonin, acetylcholine and dopamine) greatly influence neuronal circuit activity. These neuromodulators are secreted by a small group of neurons, and reach large regions of the nervous system by diffusion (see below). In addition, neurosecretory cells release neurohormones, which exert their effects on many regions of the brain via the circulatory system (Fuxe et al., 2010; Marder, 2012). Glial cells have also been proposed as having a role in information processing via bidirectional glial cell-neuron signaling (Araque et al., 2014) and it is thought that neuron-astrocyte metabolic interactions play a critical role in the coupling between neuronal activity and energy metabolism (Magistretti and Allaman, 2015). In spite of this ever-increasing complexity and massively tangled organization, it should be noted that general principles or rules for the design of brain circuits do exist. In the words of Cajal (1917) when referring to the cerebral cortex: “*Ese desorden aparente de la maraña cerebral, tan alejada de la regularidad y simetría de la médula espinal y cerebelo, esconde un orden profundo, sutilísimo, actualmente inaccesible*”. “That apparent disorder of the cerebral tangle, so different from the regularity and symmetry of the spinal cord and of the cerebellum, hides an extremely subtle, profound organization which is at present inaccessible”. Discovering these rules is clearly of critical importance. For instance, chemical axo-dendritic synapses are by far the most common type of synapse (followed by axo-somatic synapses), at least in mammals. Other types of synapses are not found in all regions of the nervous system and when they are present, they are usually only established between certain types of neurons.

A further aspect to consider is the functional significance of the various types of overall brain connectivity. For example, a wide range of functions which need information to be transmitted quickly from one point to another rely on chemical synapses as the anatomical basis for brain wiring. Reflexes are a good example of this—their neuronal circuits give rise to quick and simple actions, which proceed automatically and subconsciously. Other functions based on point-to-point synaptic wiring are not so simple, however, including the processing of information in large but discrete circuits in the motor and sensory systems, and in those regions of the brain that are involved in complex functions such as reasoning, calculation, language and writing. Modulatory systems, on the other hand, exert their effect on many areas of the brain via many different neuronal circuits, and it is this kind of diffuse action that is involved in more “general” brain states and moods, such as sleep, attentiveness and anxiety. It can therefore be concluded that, while there are exceptions and complexities such as those described above, the neuron doctrine continues to be one of the foundations on which our concept of nervous activity is based (Shepherd, 1991; Jones, 1994).

## A First Step Forward: The Connection Matrix of the Brain

As discussed above, one of the first steps towards understanding how neuronal circuits contribute to the functional organization of the brain is to define its detailed structural design and to map its connection matrix. In the words of Swanson and Bota (2010), “the wiring diagram of the nervous system’s structural connectivity provides an obligatory foundational model for understanding functional localization at molecular, cellular, systems, and behavioral organization levels”. The connectivity of the brain can be analyzed at three quite distinct levels (Sporns et al., 2005; DeFelipe, 2010):

1. Macroscopically, focusing on major tract connectivity, for example by examining images of the whole brain (or of large brain regions), which can even be performed *in vivo* by MRI or other techniques.
2. At an intermediate resolution as can be achieved by light microscopy, which also allows putative synaptic contacts to be mapped.
3. At the ultrastructural level, which can only be studied using electron microscopy and serves to map true synaptic contacts.

Thus, it has been proposed that the term “connectome” be used to refer to the map of connections at the macroscopic and mesoscopic levels and “synaptome” for the map at the ultrastructural level (DeFelipe, 2010).

Powerful methods are currently available that allow the connectome to be traced in meso- (intrinsic or local connections) and macrocircuits (long distance connections). Classical tracing methods (Jones, 2007a) can be used for this purpose, as can molecular/genetic/physiological approaches and imaging techniques, including two-photon imaging and ontogenetic techniques. The development of these techniques with the aim to include large brain structures and cell-type specificity is already providing very important advances in the knowledge of the anatomical and functional connectome in animal models (see Osten and Margrie, 2013). Furthermore, the development of retrograde and anterograde trans-synaptic tracers to directly study cell-to-cell connectivity and the combination of these tracers with *in vivo* imaging and optogenetic methods and/or inducible gene expression in transgenic mice will represent major advances in the anatomical/functional study of the neural circuits at the mesoscopic level (see Osakada et al., 2011).

Nevertheless, it should be noted that, in general, connectivity visualized at the light microscopic level is rather basic (e.g., connections between brain regions) and, in most cases, point-to-point connections between local neurons and between neurons or afferent fibers cannot be accurately determined (see DeFelipe, 2010). The reason for this is that when a given labeled axon is seen in contact with another labeled neuronal element, it does not necessarily mean that there is a synaptic junction as axonal boutons are adjacent to several possible synaptic targets of which only those that are labeled are visible. In addition, not all axonal boutons establish synaptic contacts and indeed a large proportion of certain axonal systems are non-synaptic, like the axons containing dopamine, noradrenaline, serotonin and acetylcholine. These axonal systems have been examined in several cortical areas of the rat, cat, monkey and human, and in a number of other regions of the central nervous system of the rat, showing a similar low frequency of synaptic contacts although this frequency varies between brain regions (reviewed in Descarries and Mechawar, 2000). However, the axonal boutons from other types of neurons may establish more than one synapse (multiple synapses). For example, it is relatively common to observe the establishment of multiple synapses by: (i) the axonal boutons from thalamocortical afferents—in the visual cortex of both cat and macaque (Freund et al., 1985, 1989); (ii) interneurons like double bouquet cells—in several areas of the macaque and human cerebral cortex (DeFelipe et al., 2006); and (iii) basket cells and dendritic-targeting cells—in the cat visual cortex (Tamás et al., 1997). Furthermore, the studies of White et al. (2004), using serial section reconstructions at the electron microscopic level of thalamocortical axons in mouse barrel cortex, have shown that although the vast majority of synapses are established by the axonal boutons or varicosities (88%), they also occurred at cylindrically shaped regions of the axonal segments (12%). Thus, the presence of a labeled terminal in close apposition with a given neuronal element can only be considered as a putative synaptic contact, whereas an inter-varicose segment of an axon may establish a synapse with an adjacent neuronal element. Keeping all of these points in mind, it is therefore clear that the available connectome diagrams are imprecise.

**Figure 3:**
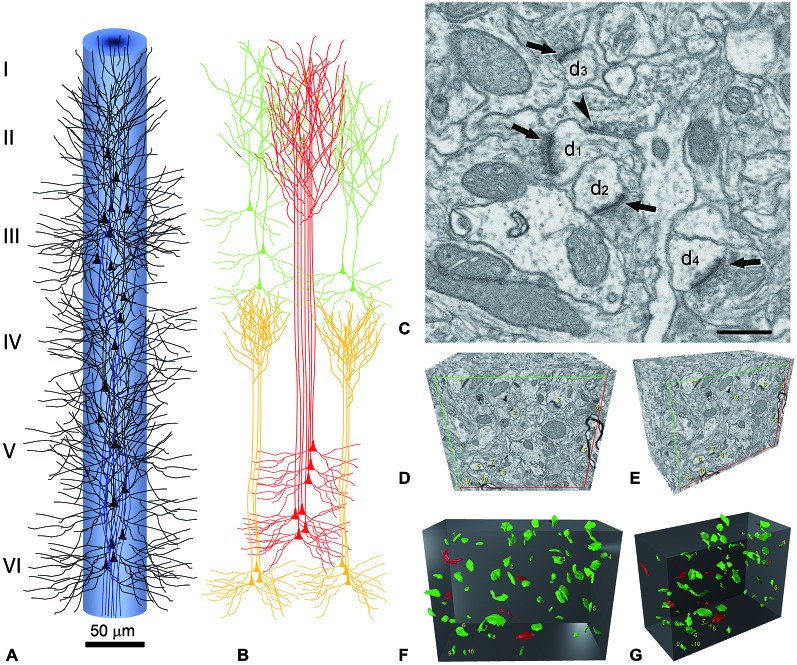
**Reconstruction of a minicolumn. (A)** Schematic representation of a minicolumn in which only the soma and proximal dendrites of pyramidal cells (black) and the main axon (blue) are represented. Note that the axons form bundles due to the vertical arrangement of pyramidal cells. **(B)** The apical dendrites also form vertical bundles and, although variability exists both between cortical areas and species in the size and number of dendrites that form the bundles as well as in the layer where the terminal dendritic tufts terminate, in general, the vertical dendritic organization is as follows. As reviewed in DeFelipe (2005), distinct bundles of pyramidal neuron apical dendrites are formed in different levels of layer V, and ascend towards the pial surface. Apical dendrites originating from pyramidal cells in layers II–III mainly join the bundle periphery. At this level, the apical dendrites of layer V and layer II–III pyramidal neurons begin to form terminal tufts which end in layer I. By contrast, the apical dendrites originating from Layer VI pyramidal cells do not join the layer V bundles, but are arranged as separate bundles which ascend to layer IV and form terminal tufts there. The core of the long dendritic bundles that extend from layer V to layers II–III is therefore principally composed of the apical dendrites pertaining to layer V pyramidal neurons. **(C)** Image captured by focused ion beam milling and scanning electron microscopy (FIB/SEM) to show the relatively high density of synapses in the neuropil and the ultrastructural appearance of asymmetric and symmetric synapses in the rat cerebral cortex. Four asymmetric synapses (arrows) and one symmetric synapse (arrowhead) can be identified on four dendritic spines (d1 to d4). Asymmetric synapses show a thick post-synaptic density. The symmetric synapse has a thin post-synaptic density, which is similar to the pre-synaptic density, and is located on the neck of a dendritic spine (d1). Scale bar, 500 nm. **(D–G)** Three-dimensional representation of a stack of serial sections and the synaptic profiles that appear in the corresponding counting brick. **(D,E)** show a stack of serial sections, slightly rotated counter-clockwise through the vertical axis in **(E)**. Only 12 sections are shown out of the 115 that compose the complete stack. An unbiased counting frame was drawn on each section, taking the green and the red lines as the acceptance and exclusion boundaries, respectively. To extend the counting frame to three dimensions, the front section was considered as an acceptance plane and the last section as an exclusion plane. Thus, synaptic profiles (contours of the synaptic membrane densities) were counted inside an unbiased counting brick bound by three acceptance planes (top, left and front) and three exclusion planes (right, bottom and back). As an example, the 10 synaptic profiles that appeared in the first section (acceptance plane), without intersecting any of the exclusion planes, have been numbered from 1 to 10 in **(D,E)**. The counting frame measured 6.86 × 5.28 μm after correction for tissue shrinkage. In **(F,G)** the counting brick and the three dimensional reconstructions of synaptic profiles have been rendered. Green objects represent asymmetric synaptic profiles and red objects symmetric synaptic profiles. All the objects shown were inside the counting brick or intersected one of the acceptance boundaries, without intersecting any of the exclusion planes. Numbered objects correspond to the same synaptic profiles shown in **(D,E)**. Note that every object can be individually identified and localized in the 3D space. Panels **(A,B)** have been adapted from DeFelipe (2005), and **(C–G)** and legend have been taken from Merchán-Pérez et al. (2009).

Electron microscopy with serial section reconstruction is the favored method for tracing the synaptome, and this technology has a proven track record for acquiring 3D data from ultrathin sections. However, it is exceedingly time-consuming and challenging to obtain long series of such sections. As a result, the reconstruction of large tissue volumes is usually not possible. The recent development of automated or semi-automated electron microscopy techniques (which require much less labor-intensive human interaction and training than conventional electron microcopy) represents an important advance in the study of the synaptome (Denk and Horstmann, 2004; Smith, 2007; Helmstaedter et al., 2008; Knott et al., 2008; Merchán-Pérez et al., 2009). For example, the 3D reconstruction method involving the combination of focused ion beam milling and scanning electron microscopy (FIB/SEM; Figures 3D–G) permits the rapid and automatic serial reconstruction of relatively large tissue volumes (Knott et al., 2008; Merchán-Pérez et al., 2009). Nevertheless, even using this FIB/SEM technology, full reconstruction of whole brains will only be possible in some invertebrates or for relatively simple nervous systems. Indeed, even for a small mammal like the mouse, it is *impossible* to fully reconstruct the brain at the ultrastructural level since the magnification needed to visualize synapses yields relatively small images (in the order tens of μm^2^).

It is perhaps useful to take the cerebral cortex as an example, and consider what would be required to fully reconstruct just one minicolumn (Figure 3)—defined as a vertical column through the cortical layers made up of the regular columnar disposition of the radial bundles of myelinated axons (radial fasciluli) or vertical aggregates of somata of pyramidal neurons or vertical bundles of apical dendrites of pyramidal cells (see e.g., Fleischhauer et al., 1972; Peters and Walsh, 1972; reviewed in DeFelipe, 2005; Rockland, 2010). In order to fully reconstruct a typical diameter of 50 μm and a height from the pial surface to the white matter of 2000 μm, using sections of 100 μm^2^ at a thickness of 20 nm, we would need 1.9625 × 10^6^ sections.

The next step would be the huge task of following each of the millions of neuronal and glial processes of the image stacks to fully reconstruct all the elements that make up the minicolumn. If this were possible, then we would have to face another big problem which is that the volume occupied by the whole dendritic and axonal arbors of neurons within the minicolumn exceeds the boundaries of this tissue volume, and consequently a substantial proportion of their dendrites and axons would be cut, in particular the axonal arborizations.

**Figure 4:**
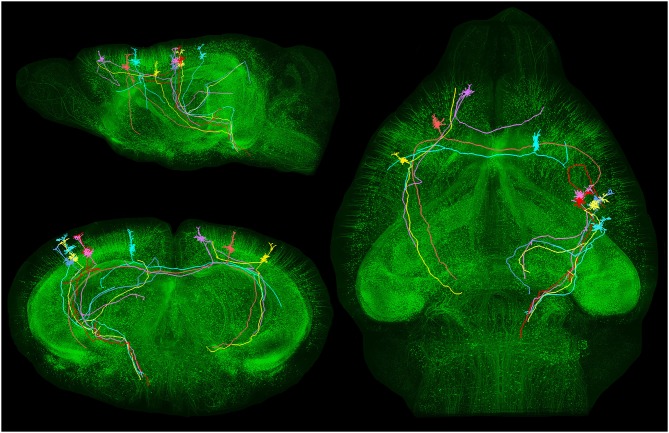
**Long-distance axonal projections of individual pyramidal neurons**. Images obtained from an adult Thy1-eGFP mouse brain using a fluorescence micro-optical sectioning tomography (fMOST) method. In this figure is shown the long-distance projectionpattern of eight layer V pyramidal neurons located in different cortical areas. 3D reconstruction results were merged with the direct volume rendering of a whole brain image stack in sagittal, coronal and horizontal views. The image stack had been resampled from a voxel size of 0.32 × 0.32 × 2 μm^3^ to 4 × 4 × 4 μm^3^. Courtesy of Hui Gong. Unpublished material taken from Gong et al. (2013).

**Figure 5:**
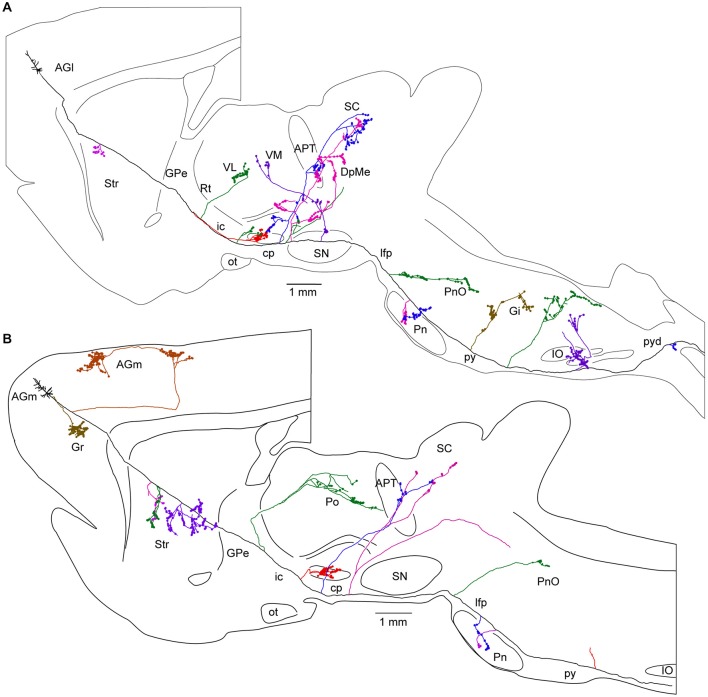
**Long-range corticofugal axons**. Tracing of single axons labeled with small injections of biotinylated dextran amine in the rat motor cortex. **(A)** Axon of a lateral agranular cortex (AGl) pyramidal tract neuron that emits multiple collaterals (shown with different colors) including subthalamic nucleus (STN) collaterals. No cortical collateral was found, though this neuron had multiple collaterals innervating striatum (Str), thalamic, mesencephalic, pontine, and medullary nuclei. The STN collaterals of the neurons had thin branches entering zona incerta (ZI). One of the cerebral peduncle collaterals of the neuron emitted ZI branch forming boutons. **(B)** Axon of a medial agranular cortex (AGm) pyramidal tract neuron that emits multiple collaterals including STN, Str, thalamic, and pontine nuclei. The neuron had cortical collaterals innervating AGm, granular cortex (Gr), and Str. The thalamic collateral of the neuron travelled through the middle of the thalamus. One of the cerebral peduncle collaterals of the neuron B traversed STN and then to ZI without forming boutons. Other abbreviations: APT, anterior pretectal nucleus; cp, cerebral peduncle; DpMe, deep mesencephalic nuclei; Gi, gigantocellular reticular nucleus; GPe, Globus pallidus external segment; ic, internal capsule; IO, inferior olive: lfp, longitudinal fasciculus of the pons; ot, optic tract; Pn, pontine nucleus; PnO, pontine reticular nucleus, oral part; Po, posterior thalamic nuclei; py, medullary pyramid; pyd, pyramidal decussation; Rt, reticular thalamic nucleus; SC, superior colliculus; SN, substantia nigra; VL, ventrolateral thalamic nucleus; VM, ventromedial thalamic nucleus. Courtesy of Hitoshi Kita. Figure and legend taken from Kita and Kita (2012).

In order to better appreciate the importance of the problem, it is sufficient to visualize the long trajectory and bifurcations of individual pyramidal cell axons across the whole mouse brain (Gong et al., 2013; Figure 4) or the complex axonal arborization patterns of single pyramidal cells in the rat brain (Kita and Kita, 2012; Figure 5).

**Figure 6:**
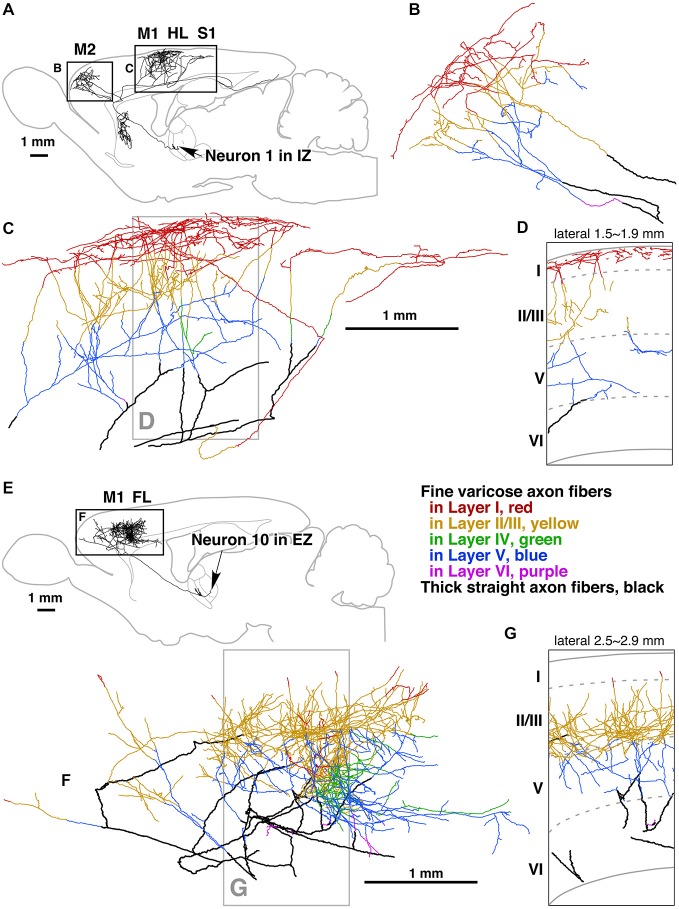
**Camera lucida reconstruction of two motor thalamocortical axons in the rat labeled with viral vectors**. Axon fibers of IZ neurons (inhibitory afferent-dominant zone of the ventral anterior-ventral lateral motor thalamic nuclei [VA-VL complex]) were widely distributed in motor-associated areas and neostriatum **(A)**. Of cerebral cortical layers, layer I was most intensely innervated by the axon fibers of IZ neurons **(B–D)**. In contrast, axon fibers of EZ neurons (excitatory subcortical afferent-dominant zone of the VA-VL complex) were found only in motor-associated areas **(E)** and distributed mainly in cortical layers II–V **(F,G)**. Panels **(D,G)** are representative planes, in which the results of 10 serial sections were superimposed onto a parasagittal plane of the fifth section. Other abbreviations: FL, forelimb region of primary somatosensory-motor area; HL, hindlimb region of primary somatosensory-motor area; M1, primary motor area; M2, secondary motor area; S1, primary somatosensory area. Courtesy of Takeshi Kaneko. Figure and legend taken from Kuramoto et al. (2009).

**Figure 7:**
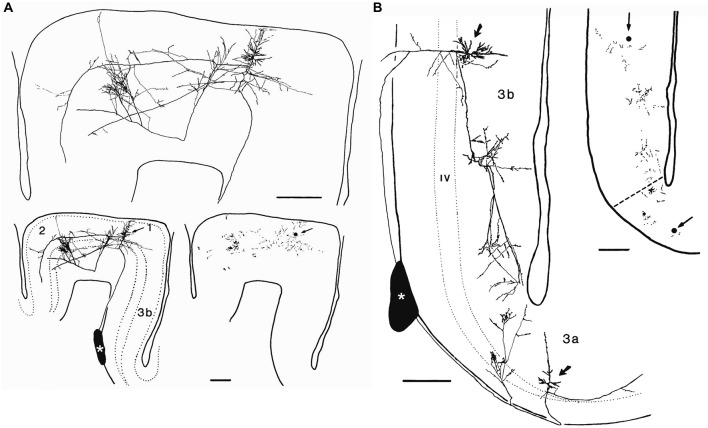
**Axonal arborizations of cortico-cortical cells in monkey sensory-motor cortex**. These neurons were labeled after small extracellular injections of horseradish peroxidase into a stratum of corticocortical axons situated in the white matter immediately deep to area 3b (asterisks). **(A)** Retrogradely labeled corticocortical cell with soma (arrow) in area 1, a minor collateral to area 3b, dense boutonal clusters in areas 1 and 2, and major collaterals apparently continuing on toward area 5. **(B)** Retrogradely labeled corticocortical cells with somata (arrows) in areas 3b and 3a and focused concentrations of boutons in each area. The boutonal plots were produced from high-magnification drawings of the full collateral ramifications. Each dot indicates one bouton. Bar, 500 μm. Taken from DeFelipe et al. (1986).

If we were to follow the extrinsic axons entering the minicolumn that establish synapses with postsynaptic elements of the minicolumn, like motor thalamocortical axons in the rat (Kuramoto et al., 2009) for instance, we would have to face a similar difficulty due to the complexity and widespread axonal arborizations of these neurons (Figure 6). This problem would be even greater if we were to follow an extrinsic axon originating in the basal forebrain. For example, the studies of Wu et al. (2014)—using genetically-directed sparse labeling to examine the full morphologies of individual basal forebrain cholinergic neurons in the mouse—have shown that individual arbors innervate multiple cortical columns, and have >1000 branch points and total axon lengths of up to 50 cm. These authors have also estimated that basal forebrain cholinergic neurons in humans have a mean axon length of ~100 meters. Furthermore, the axons of most cortical neurons (i.e., pyramidal cells) give rise to local axonal arborizations (near the cell body of origin) but the number of axonal synaptic boutons is relatively low (in the order of a few hundreds; see e.g., DeFelipe et al., 1986; Figure 7). Thus, the majority of other synapses within the minicolumn are of extrinsic origin (i.e., axon terminals coming from neurons with a distant origin, like cortico-cortical neurons, thalamo-cortical neurons, etc.). Table 1 outlines the feasibility and non-feasibility of obtaining some critical quantitative anatomical data of the minicolumn that is relevant for connectomics and models.

**Table 1:** **Feasibility and non-feasibility of obtaining some critical quantitative anatomical data of the minicolumn that is relevant for connectomics and models**.

Light microscopy		Electron microscopy	
Feasible	Non-feasible	Feasible	Non-feasible
Density and spatial distribution of neurons and glia (e.g., cells labeled with NeuN, DAPI)	^1^Volume occupied by dendrites, axons and glial processes	Proportion of different types of synapses (e.g., asymmetric and symmetric synapses)	Absolute number and distribution of different types of synapses
Density and spatial distribution of different types of neurons and glia (e.g., cells that are immunoreactive for GABA, PV, SOM, Iba1)	Inference of number of synapses established by an axon based on the assumption that one varicosity equals one synapse	^2^Identification and characterization of long-range synaptic connections	Complete synaptic connections of the axonal arborization of identified neurons
Volume fraction occupied by neurons, glia, neuropil and blood vessels	^3^Number of cells projecting to a given brain region (long-range outputs)	General identification of the postsynaptic targets to a given axon terminal (non-characterized postsynaptic dendritic shafts, dendritic spines, somata, axon initial segments)	Full identification and characterization of the postsynaptic targets (i.e., origin and characteristics of all the postsynaptic dendritic shafts, dendritic spines, somata, axon initial segments)
Detailed single cell 3D reconstructions of dendritic trees of neurons (cell filling; e.g., intracellular injections of Lucifer Yellow)	^3^Number of cells from other brain regions projecting to the minicolumn (long-range inputs)	Complete synaptic input maps on single identified neurons	^4^Absolute number of mitochondria in the neuropil
Detailed single cell 3D reconstructions of axonal arborization of interneurons (cell filling; e.g., intracellular biocytin labeling)		Spatial relationships of synapses with glial processes	
^2^Detailed single cell 3D reconstruction of axonal arborization of projecting neurons (e.g., single neuron-tracing using viral vectors)			
Surface area and volume of dendritic shafts, dendritic spines, somata and axon initial segments of identified cells (e.g., intracellularly injected cells)			
Density of different types of axon terminals (e.g., immunostained for vGlut1, vGAT)			
Complete putative synaptic maps on identified neurons (i.e., characterization of axonal boutons in apposition to neuronal somata, dendrites and axon initial segments of identified neurons			

*Abbreviations: DAPI, 4′,6-diamidino-2-phenylindole; GABA, γ-Aminobutyric acid; PV, Parvalbumin; NeuN, Neuron-Specific Nuclear Protein; SOM, somatostin; VGlut1, Vesicular glutamate transporter 1; vGAT, Vesicular GABA Transporter. ^1^This data can be inferred from the total number of neurons, glia and volume fraction of the neuropil coupled with electron microscopy data of the neuropil. ^2^This data can only be obtained in experimental animals. ^3^Only a rough estimate can be obtained and only in experimental animals. ^4^Density and spatial distribution of blood vessels coupled with the density of neurons, glia, mitochondria and synapses are fundamental data to model oxygen delivery and energy consumption of local neural circuits in order to better understand how local synaptic activity is regulated and limited by blood flow (see e.g., Blinder et al., 2013; Magistretti and Allaman, 2015)*.

In conclusion, complete reconstructions of small samples of the mammalian brain (on the nm scale) are feasible, while structures even of relatively small dimensions like the cortical minicolumns cannot be fully reconstructed. As a result, it is only possible to obtain incomplete synaptomes (DeFelipe, 2010; see also Budd and Kisvárday, 2012; da Costa and Martin, 2013).

## The Solution: Discrete Sampling and an Integrative Approach

What follows is a discussion of how to deal with the problem of imprecise connectomes and incomplete synaptomes focusing on the cerebral cortex (unless otherwise specified). Nevertheless, the proposed strategy based on a combined light and electron microscopy approach could be applied to any brain region.

While the synaptic density within a given cortical area and layer may vary, this variability remains within a relatively narrow window (DeFelipe et al., 1999). In addition, there are billions of chemical synapses but there are basically only two main types, asymmetric (excitatory; mostly glutamatergic) and symmetric (inhibitory; mostly GABAergic; Houser et al., 1984; Peters et al., 1991; Figure 3C). Other important general microanatomical rules (Feldman, 1984; White, 1989, 2007; DeFelipe and Fariñas, 1992; Nieuwenhuys, 1994; Somogyi et al., 1998; DeFelipe et al., 2002; Shepherd, 2004; Harris and Weinberg, 2012) are that the majority of synapses in the neuropil are of the asymmetric type, and that the percentage of asymmetric and symmetric synapses only varied between 80–95% and 20–5%, respectively, in all the cortical layers, cortical areas and species examined. The main postsynaptic targets of excitatory axon terminals are dendritic spines of pyramidal cells (Figure 3C), which in turn are the most abundant type of neuron (about 75–85% of the total population) and the main source of asymmetric synapses. The whole surface of the dendritic tree of pyramidal cells is covered by dendritic spines except the proximal portion (approximately 10–15 μm from the soma), which is either devoid of dendritic spines or they are found only occasionally. Moreover, the vast majority of dendritic spines establish at least one synapse (Arellano et al., 2007)—most dendritic spines establish a single synapse whereas some dendritic spines establish an additional synapse that is either asymmetric or symmetric (Figure 3C). In addition, symmetric synapses are established mainly on dendritic shafts and are the only type of synapse found on the axon initial segment, soma and proximal dendrites of pyramidal cells (i.e., the dendritic portion devoid of dendritic spines). Furthermore, the axon initial segment of most interneurons (which represent 15–25% of the total population) are devoid of synapses and their soma and proximal dendrites establish both asymmetric and symmetric synapses.

Considering the above-mentioned rules, together with the fact that it is possible to model the statistical distribution of the variation, it can be concluded that, in order to obtain the number and types of synapses present, it is not necessary to reconstruct the entire layer of a particular area. It is possible to determine the range of variability by sampling relatively small regions of the area multiple times instead. To tackle the complexity of brain organization and attempt to better understand it, the most practical approach would seem to involve integrating these data with light microscopy data, e.g., gray matter thickness, the volume fraction of cortical elements (neuropil, neurons, glia and blood vessels), neuron and glia density per volume, neuron microanatomy (i.e., patterns of dendritic arbors, distribution and density of dendritic spines, dendritic length, etc.), together with the patterns of intrinsic (intralaminar, translaminar) and long-range (cortico-cortical, thalamo-cortical, cortico-thalamic, subcortical extra-thalamic) connections (see DeFelipe, 2010). For example, for a given cortical layer, it is clearly not feasible to determine the contribution of its pyramidal cells synapses by reconstructing *all* the dendritic trees of these cells using an electron microscopy-based approach. However, an estimation of this could be achieved through the pooling of: (i) light microscopy data concerning the total cell number and their microanatomical characteristics with: (ii) FIB/SEM-derived data on axo-spinous and axo-dendritic synapse density. Another approach might be to identify and map the nature of the axon terminals, their spatial distribution etc. by using correlative light and electron microscopy combined with immunocytochemistry or other techniques to label the axon terminals (e.g., conjugate light-electron array tomography; see Collman et al., 2015). For example, it is possible to construct a variety of pyramidal cell-like elements (virtual neurons generated by modeling the quantitative morphometric measures of a given population of 3D reconstructed pyramidal cells) with realistic synaptic weights for computational models based on the morphological parameters found in real pyramidal cells and the general anatomical rules as follows:

*Number of synapses on dendrites* (N_sy_d):

N_sy_d is calculated using the following data:

L_ap = length of apical dendritic tree;
L_ba = length of basal dendritic tree;
D_sp = density of dendritic spines per μm;
Sy_sp = number of axospinous synapses (asymmetric and symmetric synapses);
Sy_sh = number of synapses on the dendritic shaft (asymmetric and symmetric synapses).

L_ap, L_ba and D_sp can be obtained from 3D reconstructions of pyramidal cells at the light microscopic level (e.g., using 3D confocal microscopy) labeled with markers that allow full visualization of their dendritic arbors (e.g., intracellular injections of Lucifer Yellow or biocytin). Sy_sp and Sy_sh can be estimated from three key synaptic rules. Rule 1 is based on the characteristics of the types and number of synapses on dendritic spines. Rules 2 and 3 are based on the proportion of the symmetric and asymmetric synapses, respectively, found in the neuropil that is on dendritic spines and dendritic shafts. These three rules are derived from electron microscopy data obtained from 3D reconstructions of the neuropil (Figures 3D–G) in the same brain tissue used to obtain the light microscopy data. In these reconstructions, the proportion of the asymmetric and symmetric synapses that are on dendritic spines and dendritic shafts can be established, as can the proportion of dendritic spines that establish one, two or more synapses and the type of these synapses. Shown below is an example of how we can apply these rules to obtain the synaptic weights based on general data obtained in several laboratories, which have not necessarily examined the same species, cortical layers or regions. The density of dendritic spines is not uniform in all regions of the dendritic arbor, and this density as well as the number of synapses on the dendrites, soma and axon initial segment might be variable—with such variability depending on the type of pyramidal cell and on the cortical layer, area, age, gender and species (e.g., DeFelipe and Fariñas, 1992). Consequently, these values should be considered as approximations of general, mean values and should be adjusted and validated in future studies, taking into account the cortical layer, area, age, gender and species examined.

The three rules are as follows:

Rule 1: All dendritic spines establish at least one asymmetric synapse, and 10% of dendritic spines form two synapses. A dendritic spine with a symmetric synapse also establishes an asymmetric synapse.

Rule 2: 31% of all symmetric synapses are formed on spines and 69% are formed on dendritic shafts.

Rule 3: 79% of all asymmetric synapses are formed on dendritic spines and 21% are formed on dendritic shafts.

Thus, the total number, types and distribution of synapses of, for example, a 100 μm length of pyramidal cell dendrite (excluding the initial portion which is free of dendritic spines) with a density of 15 dendritic spines per 10 μm of dendrite, can be estimated based on the following general estimations and assumptions:

Considering rule 1, the total number of synapses on dendritic spines is 165 (150 + 15). According to rules 1 and 2, there will be 31 × 15/100 = 4.65 symmetric axospinous synapses and 160.35 (165 − 4.65) asymmetric axospinous synapses. According to rules 2 and 3, respectively, there will be 4.65 × 69/31 = 10.35 symmetric synapses and 160.35 × 21/79 = 42.62 asymmetric synapses on the dendritic shaft. Thus, in 100 μm of dendrite there will be 160.35 asymmetric synapses on dendritic spines + 42.62 asymmetric synapses on shafts + 4.65 symmetric synapses on dendritic spines + 10.35 symmetric synapses on shafts = a total of 217.97 synapses.

*Number of synapses on the soma* (N_sy_so):

N_sy_so = Sa × Sy_so

where,

Sa = Surface area of the soma;

Sy_so = Number of synapses per μm^2^.

The surface area of the soma can be obtained from 3D reconstructions of identified pyramidal cells at the light microscopic level (e.g., using 3D confocal microscopy) from either genetically or immunocytochemically marked neurons (e.g., Thy1-eGFP-positive pyramidal cells or SMI 32-immunostained pyramidal cells, respectively). An alternative is to use pyramidal cells labeled by either retrograde tract-tracing techniques (e.g., by injecting Fast Blue) or labeled intracellularly (e.g., with Lucifer Yellow or biocytin). Sy_so can be obtained at the light microscopic level by calculating the number and density of immunoreactive puncta for GABAergic markers [e.g., GABA, the GABA transporter 1 (GAT-1), or the vesicular GABA transporter (VGAT)] found in contact with the 3D reconstructed labeled somata. Examples of this include performing immunostaining for GAT-1 in brain sections of Thy1-eGFP mice; using double immunostaining for SMI 32 and GAT-1; or combining tract tracing techniques and immunocytochemistry for GAT-1. The values obtained are then validated by partial 3D reconstruction of pyramidal cell somata at the electron microscopic level in the same brain tissue used to obtain the light microscopy data.

*Number of synapses on the axon initial segment* (N_sy_ax):

N_sy_ax = L_ax × Sy_ax

where,

L_ax = Length of the axonal initial segment;

Sy_ax = Number of synapses per μm.

The axon initial segment of identified pyramidal cells at the light microscopic level (see above) can be visualized by ankyrin G immunostaining or other markers of the axon initial segment (e.g., Antón-Fernández et al., 2015) followed by counting of the number of puncta immunostained for GABAergic markers (see above) in contact with the axon initial segment. The values obtained are then validated by 3D reconstruction of the axon initial segment at the electron microscopic level of the pyramidal cells in the same brain tissue used to obtain the light microscopy data.

Finally, the spatial distribution of the somata and the processes of the cellular components of the minicolumn to be analyzed should be taken into account in order to better interpret the electron microscopy data. For example, since the initial portion of the dendrites of pyramidal cells (approximately 10–15 μm from the soma) is free of dendritic spines (see above), most synapses observed in the 3D reconstructions of the neuropil within the minicolumn would most likely be from collateral branches of pyramidal cells originated at a distance from the minicolumn except for the apical dendritic trunks which are at the core of the minicolumn (Figure 3A). Given that it is possible to determine the length of the apical dendrites of pyramidal cells located in different layers, as well as the density of dendritic spines of the apical dendrites and the number of pyramidal cells, it would be relatively easy to obtain an estimation of the number of dendritic spines belonging to the neurons within the minicolumn. Since the number of dendritic spines within a given volume of neuropil is practically equivalent to the number of axospinous asymmetric synapses within that same volume, it would be possible to estimate how many axospinous synapses originate from the neurons within and outside the minicolumn.

In conclusion, the most appropriate route to follow at this moment in time appears to be to link detailed anatomical structural data with the incomplete light and electron microscopy wiring diagrams to build computational models as simplified abstractions, rather than attempting to fully reconstruct the cerebral cortex or any other brain region. Indeed these models are already being used to reason about the data, make predictions and suggest new hypotheses to discover new aspects of the structural and functional organization of the brain (Swanson and Bota, 2010; Kleinfeld et al., 2011; Hill et al., 2012; Helmstaedter, 2013; Morgan and Lichtman, 2013; Sporns, 2013, 2014; da Costa and Martin, 2013; Egger et al., 2014). Nevertheless, it should be kept in mind that the resulting connection matrix that could be obtained with this combined light and electron microscopy approach should be considered as a realistic statistical connection matrix. This statistical matrix does not directly correspond to the exact details of the real circuit itself since, as discussed above, variability exists in many structural (and neurochemical) aspects of the components of the circuits. In other words, all possible connection matrices are constrained by empirically based numerical rules and axonal-dendritic/somatic geometrical relationships from neuronal reconstructions, and exactly “which neuron connects with what” cannot be addressed with this approach. Thus, the functional interpretation of a given experiment based on this statistical connection matrix might not completely match the experimental findings. However, this mismatch could in fact serve to improve the wiring diagrams, making them more and more realistic by adding new connectivity principles.

## The Interdisciplinary and Collaborative Approach

It seems clear that only by combining studies at all three levels (macro-, meso-, and nano-scopic) can we fully understand the structural arrangement of the brain as a whole. However, despite the fact that neuroscience has advanced spectacularly in recent decades from genetic, molecular, morphological and physiological perspectives, the question remains as to why we are still so pessimistic about adopting this kind of combined approach. The simple reason for this is that there are enormous gaps between each of these disciplines—gaps which remain practically unexplored. This is not an easy task as it requires cooperation not only between groups of neuroanatomists with expertise in different techniques, but also close collaboration between those with expertise in quite different areas, like specialists in image analysis, data analysis, theory neuroscience, computation, molecular biology, physiology, among others. This is where large international projects come into play, the idea being to pool the efforts of multiple laboratories with different areas of expertise—coordinated through big worldwide projects like the Human Brain Project (HBP) based in the European Union and the Brain Activity Map based in the United States (Markram, 2013; Jorgenson et al., 2015; Zeki, 2015). Thanks to these and other initiatives that promote interdisciplinary collaboration and data sharing, such as the Allen Institute for Brain Research^1^ or neuroinformatic platforms like NeuroMorpho.Org (Ascoli et al., 2007) and BAMS2 Workspace (Bota et al., 2014), the tempo of the development of new technologies and new strategies to study the brain can be extraordinarily increased giving us cause for optimism.

## Conflict of Interest Statement

The Associate Editor Kathleen S. Rockland declares that, despite currently hosting a Frontiers Research Topic with the author Javier DeFelipe, the review was handled objectively. The author declares that the research was conducted in the absence of any commercial or financial relationships that could be construed as a potential conflict of interest.

## Footnotes

^1^http://alleninstitute.org/
